# Fossil evidence for trait diversification in an adaptive radiation

**DOI:** 10.1038/s41598-025-23186-6

**Published:** 2025-11-05

**Authors:** Nare Ngoepe, Salome Mwaiko, Mary A. Kishe, Giulia Wienhues, Yunuén Temoltzin-Loranca, Leighton King, Colin Courtney Mustaphi, Martin Grosjean, Willy Tinner, Blake Matthews, Hendrik Vogel, Oliver Heiri, Eliane Jemmi, Moritz D. Lürig, Mikkel Winther Pedersen, Ole Seehausen, Moritz Muschick

**Affiliations:** 1 Aquatic Ecology and Evolution, Institute of Ecology and Evolution, University of Bern, Bern, CH-3012 Switzerland; 2 Department of Fish Ecology and Evolution, EAWAG, Swiss Federal Institute for Aquatic Science and Technology, Kastanienbaum, CH-6047 Switzerland; 3 Centre for Ancient Environmental Genomics, Globe Institute, University of Copenhagen, Oester Voldgade 7, Copenhagen, Denmark; 4 Tanzania Fisheries Research Institute (TAFIRI), Dar es Salaam, Tanzania; 5 Institute of Geography and Oeschger Centre for Climate Change Research, University of Bern, Hallerstrasse 12, Bern, CH-3012 Switzerland; 6 Institute of Plant Sciences and Oeschger Centre for Climate Change Research, University of Bern, Altenbergrain 21, Bern, CH-3013 Switzerland; 7 Geoecology, Department of Environmental Sciences, University of Basel, Basel, CH-4056 Switzerland; 8 Center for Water Infrastructure and Sustainable Energy (WISE) Futures, The Nelson Mandela African Institution of Science and Technology, P.O. Box 9124, Arusha, Tanzania; 9 Knowledge Core GmbH, Basel, CH-4051 Switzerland; 10 Institute of Geological Sciences and Oeschger Centre for Climate Change Research, University of Bern, Baltzerstr. 1 + 3, Bern, CH-3012 Switzerland; 11 Florida Museum of Natural History, University of Florida, Dickinson Hall, 1659 Museum Road, Gainesville, FL 32611 − 7800 USA; 12 Present Address: Systematic Zoology, Faculty of Biology, LMU Munich, Grosshaderner Str. 2, Planegg-Martinsried, D-82152 Munich, Germany

**Keywords:** Adaptive radiation, Biodiversity, Diversification, Early burst, Explosive evolution, Morphospace expansion, Ecology, Ecology, Evolution, Zoology

## Abstract

Adaptive radiation is an important process for the origin of functional and ecological biodiversity. Understanding how, when, and why adaptive radiations occur is a long-standing interest in evolutionary ecology. Although many adaptive radiations have been studied, few studies resolved the temporal sequence of events during adaptive radiation. Here, we assembled a continuous record of tooth fossils of Lake Victoria’s haplochromine cichlid fish, the most rapid and youngest of the classical adaptive radiations, from sediment cores extending from lake refilling ~ 17 thousand years ago to the present. We use these fossil records to reconstruct ecomorphological temporal patterns in the unfolding of this adaptive radiation. Our results reveal a rapid expansion in morphospace, from an undiverse ancestral condition, within the first three millennia after the onset of the modern lake. Comparison with modern cichlid teeth suggests that large-scale diversification across the food web emerged within these first three millennia. We detect a clear signal of an evolutionary trend from trophic generalists to specialists, but we also show that generalists persisted amid the growing radiation of specialists. Altogether, this pattern confirms the unusual evolutionary potential of the Lake Victoria hybrid lineage of haplochromine cichlids that seeded the radiation and the unusual speed with which the adaptive radiation occurred.

**Supplementary Information:**

The online version contains supplementary material available at 10.1038/s41598-025-23186-6.

## Introduction

Observable patterns in biological diversity are the result of ecological and evolutionary processes acting over long periods in a non-linear fashion. Inferring rate variation is crucial for our understanding of the mechanisms of diversification, but it is challenging because extended periods need to be studied at a high temporal resolution. Most textbook examples of rapid ecological and evolutionary diversification (i.e., adaptive radiation; where a lineage diversifies into an array of species adapted to a variety of ecological niches^1^ have occurred over millions of years ago^2–4^, making it difficult to probe to test hypotheses regarding fine-scale temporal dynamics. In contrast, the Lake Victoria (LV) radiation of haplochromine cichlids occurred over extraordinarily short and recent geological time scales (the past ~ 17, 000 years)^5–7^ offering a unique window into the early stages of the process, which can be essential for testing predictions of the ecological theory of adaptive radiation^8^.

Theory suggests that phenotypic diversification in adaptive radiations follows predictable patterns^9^. One of the classical predictions is that morphological diversification begins early after colonization of a new adaptive zone, at first proceeds rapidly, but then slows down markedly as the level of diversity stabilizes (known as the early burst model)^10^. This prediction follows from the assumption that initially there are multitudes of ecological opportunities resulting from underutilized resources, which diminish as soon as the lineage diversifies and the habitat becomes saturated with species^11^. And this has been demonstrated in diatoms from ancient Lake Ohrid, where they found evidence for initial high speciation rates when there were abundant ecological opportunities^12^. An alternative, but not necessarily incompatible, conceptual model predicts diversification in stages that follow a sequence that begins with the speciation into macrohabitat specialists, followed by the emergence of trophic diversification within macrohabitats, and eventually diversification of the communication system and speciation within trophic groups^13^. For instance, a model for the African Great Lakes cichlids suggests explosive speciation began with ecological opportunity and niche-filling, followed by rapid diversification under selection and species interactions^14^. Here, we test some of these predictions directly using a detailed record of fish tooth fossils covering the entire history of the modern Lake Victoria haplochromine cichlid radiation archived in the lake sediment to reconstruct temporal and environmental correlates of phenotypic diversification.

The LV haplochromine cichlid radiation displays a wide range of trophic specializations, including species that feed on firmly attached filamentous algae, phyto- and zooplankton, insect larvae, snails, and crabs, to various types of top predators that feed on fish, their eggs or larvae (*Supp. Info. 1*). Beginning ca. 17,000 years ago, approximately 20 distinct ecological guilds have emerged in this radiation, each containing several to many species with slight variations on an overall trophic group-specific morphological theme^15,16^. However, the timing of the emergence and diversification of the ecological guilds has not been resolved. Simpson^17^ suggested that one of the dominant trends in adaptive radiation is the tendency to start from a generalized ancestral species and diversify into successively more specialized species (the generalist-to-specialist hypothesis). This trend of increasing specialization has been documented within other radiated lineages and is thought to arise from intra- and interspecific competition, leading either to the acquisition of increasingly diverse resources or to increasing finer partitioning of a given set of resources^1^.

There are strong links between the morphology of cichlid mouth parts and their trophic ecology. Diversification in the morphology of the feeding apparatus and associated behavior enables cichlid fish to exploit a wide array of food resources^18^. Variation in tooth shape within the oral and pharyngeal jaws strongly correlates with trophic ecology, reflecting morphological adaptations to different feeding strategies^15,16^. Although teeth in both jaws fulfill ecologically important and complementary functions, and the combination of both is important, outer oral teeth alone are important for the capture and initial food processing and contain important ecological information even in the absence of information on the associated pharyngeal teeth, the latter of which are important for more specialized food processing^16^. Individual teeth can unambiguously be assigned to either oral or pharyngeal jaws^19–22^. Here, we studied the shape of oral jaw teeth in sediment core fossil samples and compared them to contemporary samples to reconstruct the eco-morphological composition of cichlid communities throughout the past 17,000 years. We examined and asked if the radiation founders were generalists or specialists, and when and in which sequence new trophic guilds evolved in the course of adaptive radiation. Additionally, we investigated the patterns and rate of diversification. To the best of our knowledge, our study is the first fossil-based study of a large adaptive radiation that provides a continuous temporal record of evolutionary diversification in a key ecological trait (oral jaw tooth morphology) from the onset to the present day, allowing us to investigate patterns in the emergence and progression of an adaptive radiation.

## Ancestral and modern radiation morphospace occupation

**Fig. 1:**
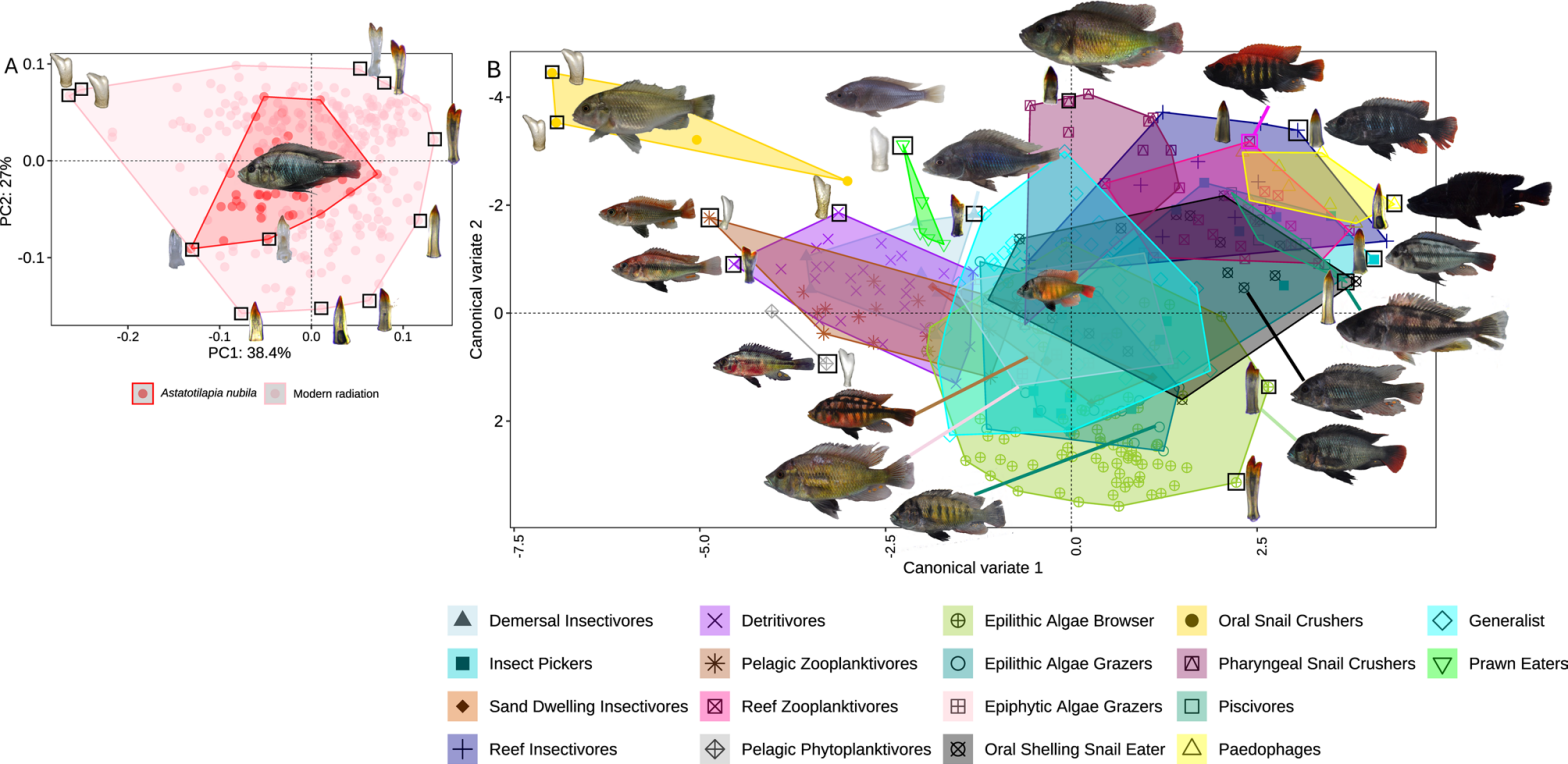
Oral tooth shape diversity of Lake Victoria cichlid fish. Patterns of morphospace occupation by the modern ecological guilds were used as the reference for interpreting the fossil data. (**A**) Principal component analysis showing morphospace occupation of the swamp-dwelling sister species of the radiation, *Astatotilapia nubila*, and species representing all the modern trophic guilds of the Lake Victoria radiation, and (**B**) canonical variate analysis showing the partitioning of total tooth shape morphospace into the modern Lake Victoria trophic guilds.

The diversity of the shape of the outer oral teeth of modern LV radiation of haplochromine cichlid fishes served as a baseline for interpreting our fossil data (Fig. 1A and B, Fig. S1), with *Astatotilapia nubila* representing the most likely morphology of the ancestral state of the modern LV radiation. *Astatotilapia nubila* is the phylogenomic sister species and closest living relative of species representing the modern LV radiation^7^ and is widespread in the swamps, wetlands and little streams around the lake. It is one of a few phenotypically similar wetland-dwelling refugial populations that survived the late Pleistocene desiccation of the lake, recolonized the lake during the early stages of refilling, and together with two other species (*A. nubila*-like generalists found in Lake Kyoga and the Western Rift lineage such as *Astatotilapia katonga*, that can be found in the Katonga river connecting Lakes Edward and Victoria over a swamp divide till this day), gave rise to a hybrid population from which the radiation subsequently emerged^7^. Our results show *A. nubila* nested within the morphospace (landmark-based geometric morphometrics) of the modern radiation, near the center of the variability of modern LV cichlids (Fig. 1A). The most generalist trophic guild from the radiation itself, and the least specialized teeth of members of more specialized guilds also occupy this position in morphospace, near the center (Fig. 1B). However, each trophic group occupies a small to moderately large part of the total radiation-wide morphospace with most overlap between trophic groups occurring in the center where *A. nubila* and the generalists of the radiation are also positioned (Fig. 1B). Meanwhile, the more specialized trophic guilds, including predators, such as piscivores and paedophages, as well as pharyngeal snail crushers, reef planktivores, reef insectivores, insect pickers, oral snail shellers and crushers, algivores, and plankton feeders, occupy the periphery of the morphospace (Fig. 1B). Significant differences between most trophic groups in oral tooth shape (Pairwise Permutational Multivariate Analysis of Variance; significance calculated using Euclidean distance P-value = 0.001) confirm that trophic groups present in the modern lake can often be distinguished from each other based on just a few teeth (Fig. 1B, *Fig. S4*,* Fig. S5*,* Fig. S6*).

### Patterns of diversification through time

**Fig. 2:**
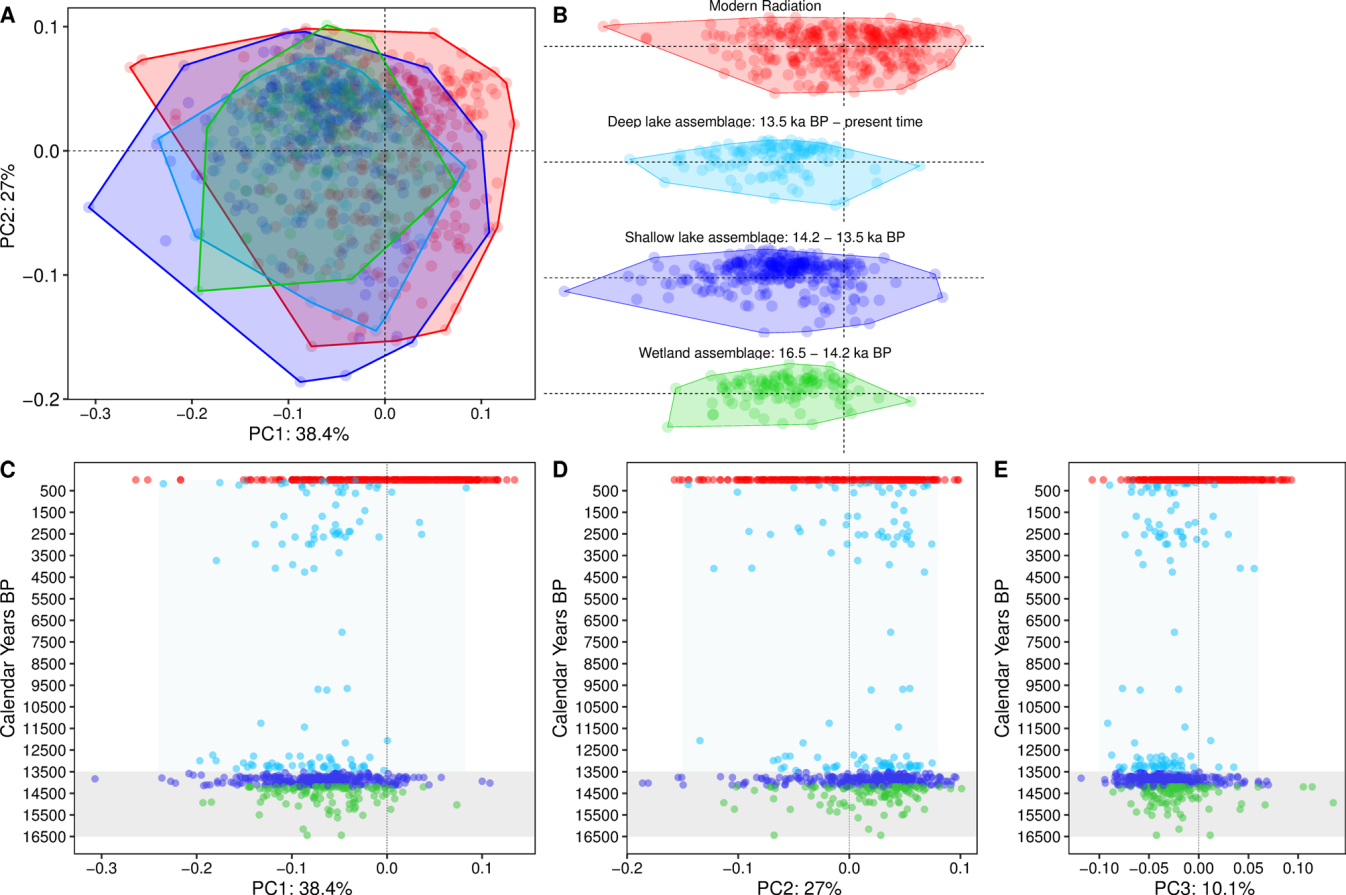
Diversification of Lake Victoria’s cichlid tooth shape through time. Patterns of morphospace occupation of the fossils binned into sequential time windows and projected into the morphospace defined by the modern Lake Victoria radiation^16^: (**A**) overlapped, (**B**) binned into sequential time intervals, followed by the distribution of fossils with (**C**) PC1, (**D**) PC2, and (**E**) PC3, along the continuous time axis from 16.5 ka BP to the present. The grey shaded area (in C-E) highlights the first 3 ka BP of the modern lake, where much of the morphological diversity emerged.

The LV basin had been completely desiccated for several thousand years until it began to fill again approximately ~ 17 ka before present (BP)^6,23^. For the first millennia of lake re-filling, it persisted as a collection of several small, shallow, swampy wetlands^6^ inhabited by haplochromine and oreochromine cichlids and an abundance of cyprinids and some catfishes^19^. As the modern large lake formed, a wealth of resources likely emerged and created multitudes of ecological opportunities that could be used by populations with large ecological versatility, potentially precursing rapid diversification provided sufficiently high evolvability in the colonizing populations^11,24^. Morphological analysis of the haplochromine cichlids’ oral tooth fossils recovered from this shallow wetland phase (~ 16.5–14.2 ka BP) shows that they occupied a relatively small area of morphospace (*Table. S1*, Fig. 2A and B); in fact, the fossil phenotypes present in this phase were not significantly different from those of the phylogenomic sister lineage of the modern species flock, *A. nubila* (dispRity pairwise *t*-tests P-value = 0.05).

In the subsequent lake phase, detailed reconstructions of terrestrial and aquatic vegetation from pollen analyses of the same cores suggest that the landscape became a mosaic of shallow lakes and extensive wetlands^6,25^. This change almost certainly opened up new aquatic habitats and resources that were not present in the preceding, occasionally drier phase. Morphological analysis of the haplochromine cichlids’ outer oral teeth fossils shows morphospace expansion from the radiation founders that occurred in the shallow swampy wetland phase (~ 16.5–14.2 ka BP) to the assemblage representing the period when the lake transitioned from the inferred wetland mosaic to an extensive but relatively shallow lake (~ 14.2–13.5 ka BP) (*Table. S1*, Fig. 2A and B). Furthermore, the tooth shape analyses show the fossil teeth phenotypes from the shallow lake environment not only significantly differed from those from wetland phase (dispRity pairwise *t*-tests P-value = 0.019), but also from *A. nubila* (dispRity pairwise *t*-tests P-value = 0.01), suggesting an increase in morphological diversity. Overall, these results suggest that the largest observed morphospace occurred within the first three millennia after the onset of the formation of modern LV (Fig. 2A-E), consistent with the LV radiation following an early burst model (Fig. 2B). This rapid trait diversification within only a few millennia is consistent with recent genetic evidence that hybridization between several *Astatotilapia nubila*-like swamp-dwelling species seeded the LV radiation when the modern lake refilled with water about 17 thousand years ago^7^. Each of these phenotypically similar generalist species was itself a product of earlier hybridization between two distantly related lineages^26^. In this scenario, the hybrid swarm provided the standing genetic variation that allowed it to diversify rapidly into many ecological niches^7^.

When the lake transitioned into a deep open habitat (from ~ 13.5 ka BP), a shift from an extensive but rather shallow lake (~ 14.2–13.5 ka BP) to a deep lake (~ 13.5 ka BP and thereafter), which is also inferred from other palaeolimnological proxies^6,23,27^, the fossil density and morphospace occupied in our offshore cored site massively declined (*Table. S1*, Fig. 2A and B). The decline does not necessarily indicate a decrease in lake-wide morphotype variability. This decline is consistent with the theory predicting that trait diversification slows down as niches fill up and progressively fewer underutilized resources are available^8^. The tooth shape analyses show a significant difference between the preceding shallow lake and this deep open lake fossil teeth phenotypes (dispRity pairwise *t*-tests, P-value = 0.017). This finding is consistent with demographic analyses of modern genomes within the LV radiation, suggesting that deep-water species evolved relatively recently within the last ~ 10 ka years^28^.

## Evolution of phenotypic diversity and ecological niche differentiation

**Fig. 3:**
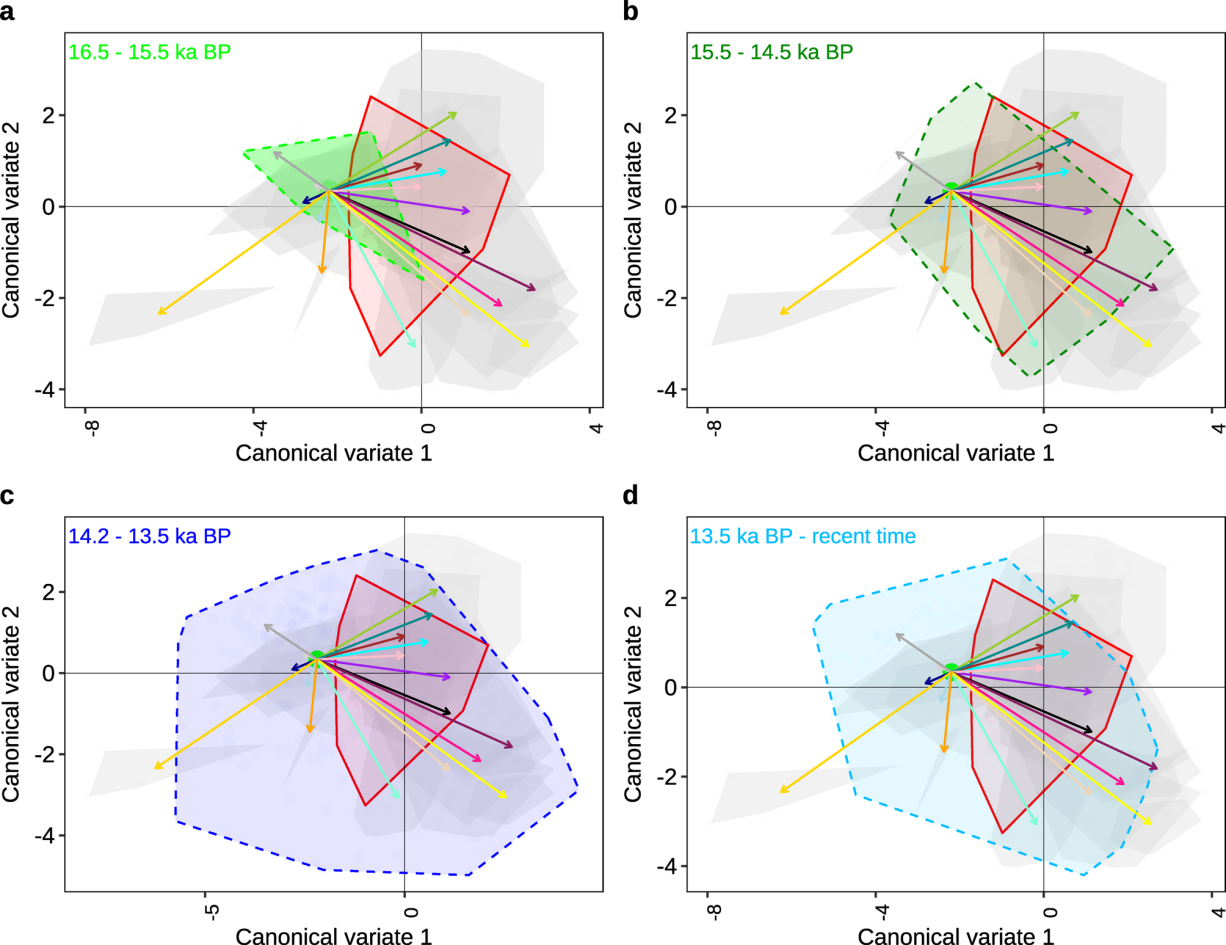
The sequence of emergence of trophic groups in the Lake Victoria cichlid fish radiation. Fossils were binned into time windows and projected into the morphospace of the modern radiation with vectors representing the direction and distance of divergence from the ancestral condition for each trophic group. Reconstruction using canonical variance analysis (**a-d**). All vectors in all time windows start from the centroid of the data points representing the fossil wetland generalist that colonized the early wetland and occupied it for at least 1000 years (~ 16.5–15.5 ka BP) before it diversified. Vectors extend to the centroids of each modern guild. The dashed polygons are the fossil-based estimate of radiation morphospace occupied in each time window, superimposed on polygons representing the modern radiation, and the red polygon is the phylogenomic sister species of the radiation, *Astatotilapia nubila*.

The tooth shape variation that we infer for the radiation founders that inhabited the shallow swampy wetland (~ 16.5–15.5 ka BP) is eco-phenotypically generalized and overlapped in morphospace (landmark-based geometric morphometrics) with the extant sister species of the radiation, *A. nubila*, that occupies shallow waters and swamps around present-day LV. This resemblance to its modern analogue applies both to the mean tooth shape and the extent of variation. From this, we infer that the haplochromines that colonized the early wetlands correspond phenotypically and likely ecologically to *A. nubila*, a classically generalized haplochromine^29^. When we use the centroid in the morphospace of the founder assemblage from the early wetland refilling phase (~ 16.5–15.5 ka BP) and relate it to the centroids of the modern trophic guilds, we can trace the direction, timing and distance of divergence away from the ancestral state and towards specialization for different ecological niches through time (Fig. 3a, *Fig. S7*). From this approach, we infer that, in the interval occurring ~ 2 ka after founding of the radiation, the assemblage began to diversify into areas of the morphospace representing novel trophic guilds. At the end of this interval, the fossil morphospace overlaps the centroids of several modern trophic guilds: generalists, pelagic zooplanktivores, pelagic phytoplanktivores, demersal detritivores and demersal insectivores. Interestingly, the fossil morphospace does not overlap the centroids of more specialized groups, like the pelagic phytoplanktivores, paedophages, piscivores, oral shelling snail crushers, epilithic algae grazers, and sand-dwelling insectivores (Fig. 3b). These results are similar whether we use canonical variance analysis (Fig. 3b) or principal component space (*Fig. S7*), with the exception of pelagic zooplanktivores. The trophic guilds covered by morphological teeth variation in this phase (~ 15.5–14. 5 ka BP) can be found in contemporary lacustrine shoreline wetlands of LV ^16^, but not all trophic guilds that can be found in such habitats were present yet in this fossil assemblage.

In the last phase of extensive shallow lakes (~ 14.5–13.5 ka BP), morphological diversity increased to a level nearly equivalent to that of the extant radiation, with centroids of all the trophic guilds recovered except the oral shelling snail crushers and most specialized epilithic algae browsers and grazers (Fig. 3c, *Fig. S7c*). Remarkably, our data suggest that most of the trophic guilds emerged within the ~ 3 ka after the onset of lake formation, suggesting rapid phenotypic and ecological diversification within just three millennia (Fig. 3). Some theoretical models predict that generalists should be favoured at the onset of adaptive radiation, and only be replaced by specialists when conditions become stable and competitive exclusion begins to act^30^. Conversely, our results show a rapid increase in the emergence of specialized phenotypes quite early in the radiation, despite the likely dynamically expanding lake environment from swamps, to connected wetlands and an extensive but shallow lake during this time^6,23,27^. Furthermore, our results confirm that from the beginning of the radiation to the present, morphological generalists continued to persist among the specialists, not only in the number of species (as known from contemporary data) but also in relative abundance. This suggests competitive exclusion may not be an important process in this particular case of adaptive radiation and corroborates the observation in Lake Tanganyika cichlids^31^ and that of Greenwood^29^ who compared members of the Lake Victoria species flock of cichlid fish. The persistence of generalists, sustained coexistence in many habitats, and the diversity of specialists indicate that top-down control and/or resource partitioning as a result of competition was central to the LV radiation, rather than outright competitive exclusion.

Morphological variation among fossil teeth younger than ~ 13.5 ka decreased compared to those from the extensive shallow lake preceding it in time (Fig. 3b and b). At that time, most coring sites became deeply inundated and from then on sampled offshore pelagic assemblages. In the modern lake, the teeth of cichlids in the offshore and pelagic species assemblages are trophically and morphologically much less diverse than those in the shallower sublittoral and littoral zones and rocky reefs. This is because the deep and pelagic habitats do not harbor species with adaptations to behaviorally complex feeding tasks involving oral manipulation of prey (epilithic browsers, grazers, oral snail shellers, oral snail crushers, insect pickers, reef insectivores, and paedophages). The morphospace regions of these trophic guilds from shallower environments were indeed not fully recovered in our fossil assemblages from the deep lake phase (Fig. 3d; *Fig. S7d*). Thus, we infer that the modern functional structure of the macrohabitat assemblages was already in place a few thousand years after the onset of the radiation. Our data confirm inferences based on molecular phylogenomic studies that most trophic guilds emerged early in the Lake Victoria radiation^7,32^.

## Generalist ancestors and specialized descendants hypothesis

The teeth obtained in the earliest wetland phase (~ 16.5–15.5 ka BP), when the lake began to deepen, were phenotypically similar to those of the modern swamp-dwelling cichlid, *A. nubila*. This suggests the phenotypic diversity within the founders was largely confined to the diversity we can now observe in individual populations of these swamp dwellers. *A. nubila* are well-known generalist cichlids, highly adaptable to a variety of habitats, and they feed on nearly all resources available, but are behaviourally and functionally unspecialized feeders^16,33^. Therefore, the overlap in morphospace of the fossil radiation founders (~ 16.5–15.5 ka BP) and modern *A. nubila* suggests the former were likely habitat generalists and unspecialized feeders as well (Fig. 3a, *Fig. S7a*). This observation is consistent with patterns of cichlid radiations shown to have been initiated by generalist riverine colonizers which were capable of inhabiting a variety of lake environments^34^. Previous research showed that *A. nubila* did not form the basis of the radiation alone. Instead, it colonized the emerging lake and encountered and hybridized with at least two other ecologically and phenotypically similar lineages that probably arrived from different wetland habitats^7^.

Upon encountering each other, these lineages merged into a single genetically variable hybrid population, and it is this hybrid population that subsequently radiated into all the trophic guilds. It seems likely that this merging of lineages would not have happened until the mosaic of shallow lakes was sufficiently extensive to bring refugial populations together from opposite sides of the lake. Only when the landscape transitioned from the early wetland condition to an extensive mosaic of shallow lakes and wetlands (~ 15.5–14.5 ka BP) did feeding specializations become apparent (Fig. 3). Subsequently, more specialized trophic groups emerged when a large but relatively shallow lake formed (~ 14.5–13.5 ka BP), the conditions in which were probably similar to those in the littoral and sublittoral of LV today. These habitats consistently host the greatest density and greatest trophic diversity of cichlid fishes in LV. The fossil record from the deep water-sampling coring sites in the late Holocene (4.5 ka BP to present time) shows significantly different phenotypes than the ancestral swamp-dweller (*A. nubila*) (*Fig. S8*). This is in line with genomic studies suggesting the deep water habitat specialists evolved around that time^7^. Overall, these observations align well with the “generalists ancestors, specialists descendants” hypothesis of adaptive radiations^1,17^.

## Concluding remarks

Our study reveals patterns of diversification in an exceptionally rapid adaptive radiation at a relatively high temporal resolution of hundreds of years. We show a burst of diversification into many distinct trophic specialists within the first three millennia of the radiation and even before the modern deep lake conditions were fully established. Our data confirms that the radiation started from a trophic generalist but does not support the notion that generalists are favored only at the onset of adaptive radiation and are replaced by specialists when conditions become stable. Instead, our findings show that trophic radiation happens in a dynamic environment and generalists persist alongside specialists. Finally, our findings add substantially to the understanding of the timing at which novel trophic guilds can evolve in cichlid fish adaptive radiations. Overall, our results confirm the unusual evolutionary potential of the hybrid lineage of haplochromine cichlids that seeded this and several other radiations in the region^7,26^.

## Methods

### The study area

The sediment cores were collected in 2018 from four sites; LVC18-S1 (located at 01°06,914’ S, 33°55,146’ E), LVC18-S2 (located at 01°07,850’ S, 33°56,780’ E), LVC18-S3 (located at 01°06,914’ S, 33°55,146’ E) and LVC18-S4 (located at 01°02,966’ S, 33°47,768’ E) in Shirati Bay area, Lake Victoria, East Africa (Fig. S1). The sites are referred to as LV1, LV2, LV3, and LV4, respectively. The cores were collected along a transect of increasing water depths and distance from shore (Fig. S1) to allow tracking of the productive and predictably most biodiverse littoral zone through time while also studying the transition from wetland refill through the shallow lake stage to the deep lake habitat within sites. Cores were taken using a UWITEC platform with metal floats, which were transported to the sites by TAFIRI’s *RV Lake Victoria Explorer* and positioned using four large steel anchors. A Niederreiter-type piston-corer with a 3 m drive length, 60 mm liner diameter, motorized hammer and hydraulic core catcher was suspended from the platform. Parallel holes with contiguous drives were cored in sites LV1, LV2, and LV3, and a series of overlapping drives from adjacent holes in LV4 ^23^. Cores were cut into ~ 1 m long sections, sealed, labeled and refrigerated during transport and storage. The cores were subsampled contiguously at 2 cm intervals, and the samples were then wet-sieved through stacked 200 μm and 100 μm mesh-size sieves to retain fish bones, scales and teeth as reported elsewhere^6,19,35^. A ZEISS stereo-microscope Stemi 508 at 10x magnification (Carl Zeiss, Heidelberg, Germany) was used to screen and sort subsamples for fossils. We created a digital photograph catalogue of modern teeth from several species of each of the present-day trophic guilds of modern haplochromine cichlids of Lake Victoria (Fig. S1). The recovered fossils were individually photographed and a detailed record with images and notes was created, followed by visual analysis using literature and our reference collection of modern teeth to assign each fossil to a taxonomic group. The sediment volume and accumulation rates, sediment chronology, age models, and lake level changes were adopted from^6,23^ with a summary in (*Supp. Info. 2*).

### The Binning of sequential time windows

#### Patterns of diversification

We created three bins of fossil teeth based on time, with the first time window referred to as the “wetland assemblage”, which begins at the onset of the modern lake at ~ 16.5 and ended at ~ 14.2 (number of haplochromine outer row teeth; *n* = 107). This bin was set to cover the period of many small wetlands and ends where the lake level reconstructions inferred the end of the wetland period ~ 14.2 ka BP ^6,24^. The second time window is referred to as the “shallow lake assemblage” (*n* = 298 haplochromine outer row teeth) and contains fossil teeth from three sites (LV4, LV1, and LV2). It starts at ~ 14.2 and ends at 13.5 ka BP, spanning the period when the lake expands to our most onshore site LV3 with evidence of a beach-like sandy layer^6,23^. The third time window was referred to as the “deep lake assemblage” and contains fossil teeth from all cored sites and all habitats (littoral to offshore; *n* = 112 haplochromine outer row teeth). This window starts from ~ 13.5 ka BP and stretches to the present time. Around 13.6 ka BP, there was a final lake rise and outflow^6,23^, marking the beginning of the modern lake with the coring sites as deep open-water habitat, a habitat that had no analogue in the old stream and wetland landscape. We also had one bin from the reference teeth from modern species (*n* = 310 haplochromine outer row teeth) representing the modern radiation with 18 trophic guilds (Fig. 1b; *Fig. **S1*).

#### Evolution of phenotypic diversity and ecological niche differentiation

The first three millennia at the onset of LV are characterized by dynamic landscape and lake level changes, necessitating a high-resolution reconstruction for the phenotypic and ecological niche differentiation (which we set at ~ 1k time bins at the beginning of the modern LV). We created four bins at one thousand years resolution at the beginning of diversification with the fossil of the first time window referred to as the “early wetland refill” which started at the onset of the modern lake at ~ 16.5 and ended at ~ 15.5 ka BP spanning the first thousand years of the modern lake (number of haplochromine outer row teeth; *n* = 6). This bin was set to cover the period when the lake bed had started to refill only in the deepest and most offshore site, LV4. The habitat was shallow water and wetland conditions, characterized by abundant *Typha* (~ 5–15%) and moderate macrophyte (e.g. *Lemna*,* Potamogeton*) pollen^6,23^. The second time window is referred to as the “wetland assemblage” phase (*n* = 40 haplochromine outer row teeth) and includes parts from three sites (LV4, LV1 and LV2). It starts at ~ 15.5 ka BP and ends at 14.5 ka BP, spanning the second thousand years of the modern lake. The water reached LV1 and LV2 during this period, with characteristic wetland fish communities and *Typha* pollen in LV2, while LV4 experienced decreasing *Typha* (~ 2–5%) and macrophyte pollen in response to increasing lake level^6,23,25^.

The third time window is referred to as the “shallow lake assemblage” (*n* = 359 haplochromine outer row teeth) and contains fossil teeth from three sites (LV4, LV1, and LV2). It starts at ~ 14.5 and ends at 13.5 ka BP, spanning the third thousand years of the modern lake. During this period, the lake expanded to our most onshore site LV3 with evidence of a beach-like sandy layer^6,23^. Seemingly, the three offshore sites corresponded to a littoral or sublittoral habitat at that time, with disappearing macrophytes and collapsing *Typha* (< 1%) at ~ 13.5 ka BP at most profundal LV4. The fourth time window was referred to as the “deep lake assemblage” and contains fossil teeth from all cored sites and all habitats (littoral to offshore; *n* = 112 haplochromine outer row teeth). This window starts from ~ 13.5 ka BP and stretches to the present time. Around 13.6 ka BP, there was a final lake rise and outflow^6,23^, marking the beginning of the modern lake with the coring sites as deep open-water habitat, a habitat that had no analogue in the old stream and wetland landscape.

Finally, we used the reference teeth from modern species (*n* = 310 haplochromine outer row teeth) representing the modern radiation with 18 trophic guilds (Fig. 1b; *Fig. **S1*). This reference collection comprises all larger guilds but lacks four small guilds: parasite eaters (two extinct species, morphologically similar to algae scrapers, ^16^), prawn eaters (nine species, where all extinct except for only one still found in the lake), scale eaters (a single extinct species with distinct teeth) and crab eaters (two species that resemble piscivores in their tooth shape^16^.

### Analysis of tooth shape

The dataset includes 511 fossil teeth and 310 modern teeth from the outer oral tooth row of species representing all trophic groups. To capture the frontal aspect of tooth shapes, 7 landmarks and curves represented by 38 evenly spaced landmarks were placed on teeth images (*Fig. S3*) using tpsDig2 software^35^. The digitized landmark coordinates were imported into Rstudio version 4.3.1^36^ and a generalized Procrustes analysis was carried out to translate and rotate the landmark configurations. Initially, we converted the reference tooth coordinates into a covariance matrix to identify the major axes of tooth shape variation in the extant radiation and reduce dimensionality using Principal Components Analysis (PCA), plotted as morphospaces using the R package Geomorph^37^. Subsequently, we projected the fossil teeth into the morphospace defined by PCA axes based on the morphospace determined by modern LV cichlid species. The package ggplot2 was used to visualize and plot shape changes along principal component axes (PC1-PC3). First, we calculated the volume of morphospace occupied to assess whether it expanded or contracted over time. To quantify phenotypic differences, we then used the R package dispRity^38^ and applied it to principal component scores (PC1–PC4), which account for approximately 75% of the total variation. Sequential disparity t-tests with Holm-Bonferroni correction were conducted across defined time windows to evaluate changes in morphological disparity through time. The package takes into account sample number variability via bootstrapping the dataset (resampling with replacement)^39^.

To visualize the morphospace occupied by different trophic guilds, we used canonical variate analysis (CVA) as implemented in the package Morpho^39^. We followed the same approach above where we reduced dimensionality using PCA and defined the axes with the reference tooth coordinates and performed CVA (using 13 PC dimensions) of the reference, followed by projection of the fossil time windows. We performed a permutational multivariate analysis of variance (PERMANOVA)^40^ to test for overall morphological differences between the groups and further conducted pairwise comparisons between the groups. To infer the sequence in which novel trophic guilds emerged from the generalized ancestor in the millennia after colonization, we projected the tooth fossils from each time slice into the reference-defined morphospace. We calculated vectors from the centroid of the fossil assemblage of the first 1000 years (representing the fossil wetland generalist) towards the centroids of the various trophic guilds as obtained from our modern reference collection. We then examined which guilds’ morphospace gets recovered by the expanding fossil assemblages and at what time. The exact position of the centroid of the fossil wetland generalist depends on the projection method into morphospace. Whereas CVA is slightly better at differentiating the trophic groups, PCA generates an unbiased positioning of individuals in overall morphospace. As such, it represents the overall variation in the ancestral population (fossil wetland generalist) and its position in the radiation morphospace. Independent of the method, the fossil wetland generalist population does not fully overlap with any of the modern trophic groups, except the generalist insectivores in the PCA-based approach (*Fig. S7*). To understand the phenotypic diversity and ecological niche differentiation, we used the CVA and PCA scores to calculate the group centroids of each trophic guild and the first-time window of the “early wetland refill” (~ 16.5 – ~15.5 ka BP). We use the wetland refill as the starting point of diversity and the trophic guilds centroids to represent the direction and distance of divergence. To finely trace the trophic guilds in the late Holocene in the deep-water lake, a bin was created from 4.5 ka BP – present time. To test for morphological differences through time we used PC scores and calculated the disparity (sum of variances) with the R package dispRity^38^ on the four bins; (1) spanning the lake wetland period from lake refill reconstructions (16.5–14.2 ka BP), (2) spanning the shallow lake period, (3) spanning the deep open lake period (13.5 ka BP – present time), and (4) modern lake fish teeth.

## Supplementary Information

Below is the link to the electronic supplementary material.

Supplementary Material 1

## Data Availability

All the data generated and analyzed in the current study, including the code to process the data and reproduce all the figures presented here, are available at figshare repositories 10.6084/m9.figshare.29254004.
