# Supplementary material for: Fossil evidence for trait diversification in an adaptive radiation
Source: Sci Rep. 2025 Nov 5;15:38824. doi: 10.1038/s41598-025-23186-6 (PMC12589450; doi:10.1038/s41598-025-23186-6)
Supplement: Supplementary file 1 — Supplementary Material 1 [file 41598_2025_23186_MOESM1_ESM.docx]

**Supplementary information**

[**Supplementary Information 1:** The teeth of trophic guilds of haplochromine cichlids in Lake Victoria](https://docs.google.com/document/d/1asYp1ykEXnRWdkJWIen2r8q8RXqikQaU9eex9_mcEzk/edit#heading=h.rvyywh8nkon)

[**Supplementary Information 2:** A map showing the study area](#_8b2xxfv1j05j)

[**Supplementary Information 3:** Sediment core geochronology and lake level variability](#_27dcj0gha0mh)

[**Figure S1:** The tooth phenotypes from modern haplochromine cichlid species used as a reference to assign fossils to trophic groups and to define the present-day morphospace.](https://docs.google.com/document/d/1asYp1ykEXnRWdkJWIen2r8q8RXqikQaU9eex9_mcEzk/edit#heading=h.bjhvo4nj48yl)

[**Figure S2:**](https://docs.google.com/document/d/1asYp1ykEXnRWdkJWIen2r8q8RXqikQaU9eex9_mcEzk/edit#heading=h.87b3jt69ycqz) A map showing t[he study area and sediment core locations.](#_8b2xxfv1j05j)

[**Figure S3:**  The outer oral teeth of haplochromine cichlids with red dots showing landmarks and curves.](https://docs.google.com/document/d/1asYp1ykEXnRWdkJWIen2r8q8RXqikQaU9eex9_mcEzk/edit#heading=h.ujj2uqn50ayq)

[**Figure S4:** Principal component analysis showing patterns of morphospace occupation by the modern Lake Victoria trophic guilds.](https://docs.google.com/document/d/1asYp1ykEXnRWdkJWIen2r8q8RXqikQaU9eex9_mcEzk/edit#heading=h.loccn467g687)

[**Figure S5:** The matrix of statistical significance of pairwise differences between trophic guilds from principal components analysis.](https://docs.google.com/document/d/1asYp1ykEXnRWdkJWIen2r8q8RXqikQaU9eex9_mcEzk/edit#heading=h.4sgtkmcf7ff1)

[**Figure S6:** The matrix of statistical significance of pairwise differences between trophic guilds from canonical variance analysis.](https://docs.google.com/document/d/1asYp1ykEXnRWdkJWIen2r8q8RXqikQaU9eex9_mcEzk/edit#heading=h.bx0wjln5dpe)

[**Figure S7:** Sequential emergence of trophic groups in the Lake Victoria cichlid fish radiation.](https://docs.google.com/document/d/1asYp1ykEXnRWdkJWIen2r8q8RXqikQaU9eex9_mcEzk/edit#heading=h.ipdoodzte0s)

[**Figure S8:** A principal component analysis of the fossils from the deep lake assemblage and the swamp ancestor (*Astatotilapia nubila*).](https://docs.google.com/document/d/1asYp1ykEXnRWdkJWIen2r8q8RXqikQaU9eex9_mcEzk/edit#heading=h.ctq9tgij6icy)

[**Table S1:** Sequential disparity analysis from one-time window to the next.](https://docs.google.com/document/d/1asYp1ykEXnRWdkJWIen2r8q8RXqikQaU9eex9_mcEzk/edit#heading=h.m7a8hdujks1d)

[**Supplementary References**](https://docs.google.com/document/d/1asYp1ykEXnRWdkJWIen2r8q8RXqikQaU9eex9_mcEzk/edit#heading=h.a7ycdydrw4f)

# **Supplementary Information 1: The trophic guilds in Lake Victoria**

**Generalists**

The generalist cichlids are highly adaptable to a variety of habitats, thus they are distributed across a wide range of habitats in the lake and feed on nearly all resources available but only absent in pelagic and profundal deep waters. They are relatively unspecialized feeders with the outer row oral teeth usually bicuspid. The species representing this trophic group include *Astatotilapia nubila, Paralabidochromis* “rock kribensis” and *Paralabidochromis* “short snout scraper” [^1^](https://paperpile.com/c/wnejhS/45n5Y)*.*

**Phytoplanktivores**

Species of this trophic group are largely found in the pelagic. They feed on phytoplankton, including blue-green algae, diatoms, and green algae. The outer row of oral teeth is usually bicuspid, and pharyngeal teeth are fine and hooked to comb aggregates of mucus and phytoplankton into the esophagus. Species representing this trophic group include *Haplochromis phytophagus* and *Haplochromis erythrocephalus* phytoplankton eaters [^2^](https://paperpile.com/c/wnejhS/bSD75).

**Epilithic Algae Grazers**

Species of this trophic group are rock dwellers and are found in areas with rock habitat in the lake. They are major consumers of sessile algae on the rocks and also feed on some insects. The outer rows of oral teeth are sub-equally bicuspid and algae-grazing fish have large mouths that they open wide and press against the rock surface to harvest loose algae. *Haplochromis nigricans* is a predominant epilithic algae grazer [^2^](https://paperpile.com/c/wnejhS/bSD75).

**Epiphytic Algae Grazers**

Most species of this trophic group occur in shallow littoral regions with emergent vegetation. They feed largely on diatoms and insects, and occasionally on blue-green and green algae. The outer row of oral teeth is bicuspid with a large flange on the major cusp. Since epiphytic algae are found on the surface of submerged vegetation, the grazers scrape them from leaves and hold them between their jaws. Species representing this trophic group include *Haplochromis nuchisquamulatus, H. obliquidens and H. lividus*  [^3^](https://paperpile.com/c/wnejhS/83Bms).

**Epilithic Browser**

The species of this trophic group are characterized by biting and tearing off algae from the substrate. They are found in rocky and aquatic plant regions of the lake. The outer row of oral teeth are bicuspid and tricuspid which are densely packed, especially on the lower jaw. Since algae attached to rock surfaces are tough, usually the fish in this trophic group shake their body with jaws attached to the surface to tear off the algae. Several species represent this trophic group and are specialized biters of filamentous algae [^5^](https://paperpile.com/c/wnejhS/8fFFB) including *Neochromis gigas, Neochromis omnicaeruleus, Neochromis rufocaudalis* [*^4^*](https://paperpile.com/c/wnejhS/IJVoU) and *Neochromis* “unicuspid scraper”.

**Plant Eaters**

The plant-eater species are found in shallow littoral regions with rooted aquatic plants. They feed on phanerogam tissue and epiphytic diatoms and algae. The outer row of oral teeth are bicuspid with a large flange, infrequently with long and slender unicuspid teeth. *Haplochromis acidens* is a well-known herbivore with a relatively short intestine adapted to a plant-based diet [^6^](https://paperpile.com/c/wnejhS/vODyr).

**Detritivores**

The detritivores are the most common trophic group of the haplochromine cichlids. They are found just above muddy bottoms in deepwater and sub-littoral regions of the lake. They feed on bottom debris and that includes decomposing plants and other remains. The outer row of oral teeth is generally unequally bicuspid. Species representing this trophic group include *Haplochromis paropius* and *H. erythrocephalus* [^2^](https://paperpile.com/c/wnejhS/bSD75).

**Insectivores**

The insectivore species are distributed in all areas of the lake including muddy bottoms in the sublittoral regions. Some species in this trophic group are morphologically and dentally generalized fish, but there are also highly specialized feeders. They feed on insect larvae and pupae and feed by sucking or picking the prey. The outer row of oral teeth is bicuspids (stout) and unicuspids (straight or slightly curved). One of the most specialized insectivores is *Paralabidochromis chilotes* which has unicuspid and slightly curved teeth that allow for extracting larvae from burrows and holes in rocks. Other species representing this trophic group include *Pundamilia macrocephala* and *P. pundamilia*, and the more generalized type *Haplochromis emodisma* [^3^](https://paperpile.com/c/wnejhS/83Bms).

**Oral Shelling Snail Eaters**

The oral sheller species are found at the bottom of the lake in sandy littoral, on rocks, and in sublittoral habitats. They feed on gastropods, bivalves, and insect larvae. The outer row of oral teeth are stout, unicuspid, acutely pointed, and (strongly) recurved. The oral shelling species includes *Haplochromis xenognathus* and the species removing snails from the shells *H. prodomus* [^3^](https://paperpile.com/c/wnejhS/83Bms).

**Pharyngeal Snail Crushers**

The pharyngeal crushers are gastropod predators found at the bottom of the lake's overall substrates. They feed on gastropods, other mollusks, and occasionally on insect larvae. The outer row of oral teeth are stout and unicuspid (blunt) and/or bicuspid. The pharyngeal teeth are molariform and the jaws are enlarged to enable the fish to crush gastropod shells. The species exclusively crushing and feeding on mollusks include *Haplochromis ishmaeli* and *H. pharyngomylus* [^2^](https://paperpile.com/c/wnejhS/bSD75).

**Piscivores**

Piscivores are at the top of the food pyramid and predate primary, secondary, and tertiary consumers. The species of this trophic group are distributed in all areas of the lake, including shallow littoral regions and pelagic zones with a few in rocky habitats. They feed on haplochromine cichlids with specialization on different prey sizes. The outer row of oral teeth are strong unicuspid (dominating) and occasionally weakly bicuspid teeth. Fine and strong upper and lower pharyngeal teeth provide a macerating action to move caught prey with ease. Many species represent this trophic group, including *Haplochromis bartoni*, *H. gilberti, H. guiarti,* *H. pellegrini* [^3^](https://paperpile.com/c/wnejhS/83Bms) and *H. serranus*.

**Zooplanktivores**

Zooplanktivores are the second most common trophic group of the haplochromines [^7^](https://paperpile.com/c/wnejhS/goSO3). There are two main groups, the pelagic and reef dwellers. They are distributed in all areas in the lake — from littoral, sublittoral and deepwater regions. They largely feed on zooplankton (cycloid copepods, calanoid copepods, and cladocerans), also *Chaoborus* larvae and pupae during the night. The outer row of oral teeth are unequally bicuspid and unicuspid in a few species. The reef zooplanktivores species include *Pundamilia nyererei* and *Lithochromis* sp. ‘yellow chin’ *pseudonigricans*, the pelagic zooplanktivores species *Yssichromis laparogramma* and *Yssichromis pyrrhocephalus* [*^2^*](https://paperpile.com/c/wnejhS/bSD75).

**Scale Eaters**

The scale eaters are largely benthic species over mud, sand, and rocky bottoms. They largely feed on scales, diatoms, phanerogam tissue, and occasionally on insects. The outer row of oral teeth are bicuspid with an expanded crown. A scale scraper *H. welcommei* shows quite stout and recurved bicuspid outer row teeth used to scrape scales from the caudal fins of cichlids [^3^](https://paperpile.com/c/wnejhS/83Bms).

**Parasite Eaters**

The parasite eaters, also referred to as cleaner fish are found over muddy bottoms. They feed on carp lice and leeches, small amounts of zooplankton, and diatoms. The outer row oral teeth are unequally bicuspid teeth with an obliquely truncated major cusp. The species of this trophic group, *H. teunisrasi* and *H. cnester*, usually have slightly curved heads [^3^](https://paperpile.com/c/wnejhS/83Bms).

**Crab Eaters**

The one species included in this trophic group is found on rocky outcrops and rocky islands. It feeds on crabs, juvenile haplochromine cichlids, prawns, and insect larvae. The outer row oral teeth are moderately recurved, and acutely pointed unicuspid. *H. howesi*, also known as *H.* sp. “smoke” is the only known species of this trophic group [^1–3^](https://paperpile.com/c/wnejhS/bSD75+83Bms+45n5Y).

**Prawn Eaters**

The species of this trophic group are largely benthic and occur in the muddy bottoms of the lake. They generally occur in low densities and feed on prawns i.e. *Caridina nilotica* and sometimes on zooplankton as well. The outer row of oral teeth are pointed unicuspid (and/or bicuspid) and tricuspid teeth. Species representing this trophic group include *H. cryptogramma* and *H. tridens*  [^3^](https://paperpile.com/c/wnejhS/83Bms).

**Paedophages**

The species of this trophic group are specialized in preying on eggs and embryos of other haplochromine cichlids. Two approaches of paedophage species are known; one is associated with forcefully engulfing the snout to obtain eggs and larvae from the mouthbrooding haplochromine cichlid females, and the other snatches the eggs from the substrate during spawning. The outer row of oral teeth are weakly bicuspids and unicuspids that tend to be buried in the thick oral mucosa. *H. melanopterus* [*^1^*](https://paperpile.com/c/wnejhS/45n5Y) and *H. maxillaris* are some of the species representing this trophic group [*^2^*](https://paperpile.com/c/wnejhS/bSD75).

**
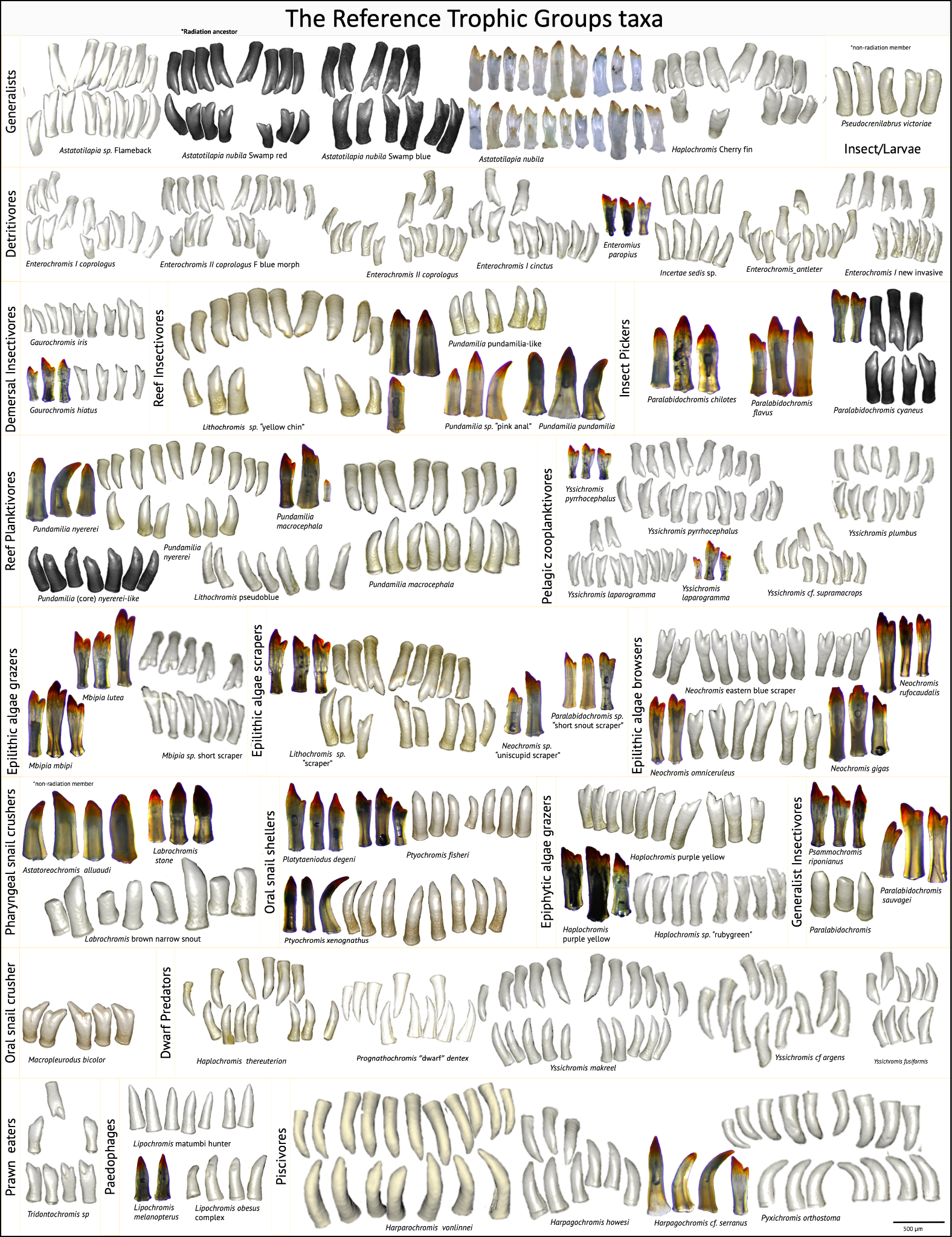
**

**Figure S1:** The tooth phenotypes from modern haplochromine cichlid species used to define the present-day morphospace and the distribution of guilds within it.

# **Supplementary Information 2: The study area**

The sediment cores were collected in 2018 from four sites in the Shirati Bay area, Lake Victoria, Tanzania, Eastern Africa (*Fig. S2*).

**
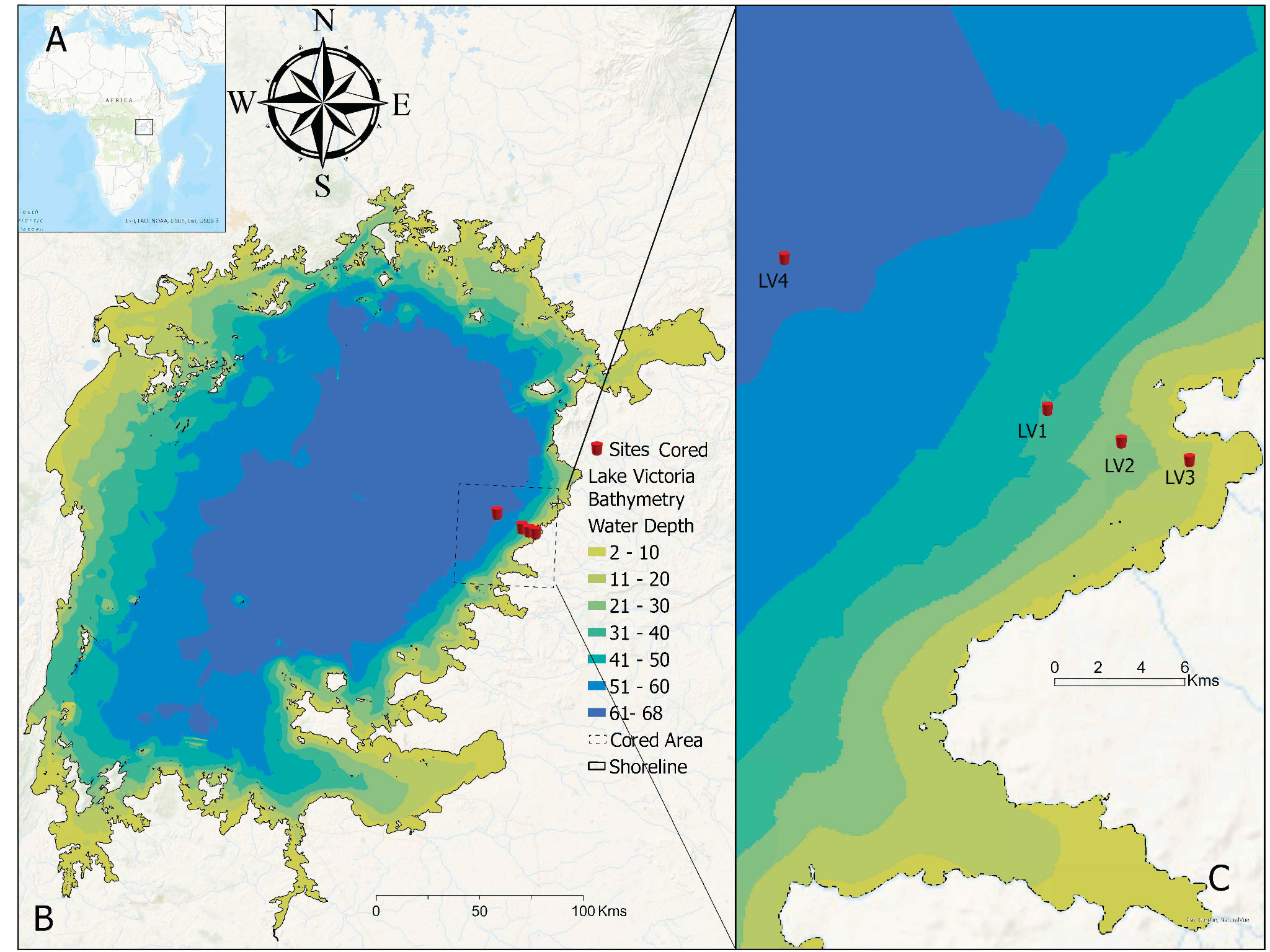
**

**Figure S2:** (A) African continent inset map with the box showing Lake Victoria. (B) Lake Victoria map with isobaths [^8^](https://paperpile.com/c/wnejhS/dMsYC) of 10 m increments. (C) The coring sites from deep to shallow (LV4 at 63 m water depth, LV1 at 37 m, LV2 at 22 m, and LV3 at 13 m) and offshore to inshore distances offshore (LV4 at 30 km, LV1 at 10 km, LV2 at 6 km, and LV3 at 2 km).


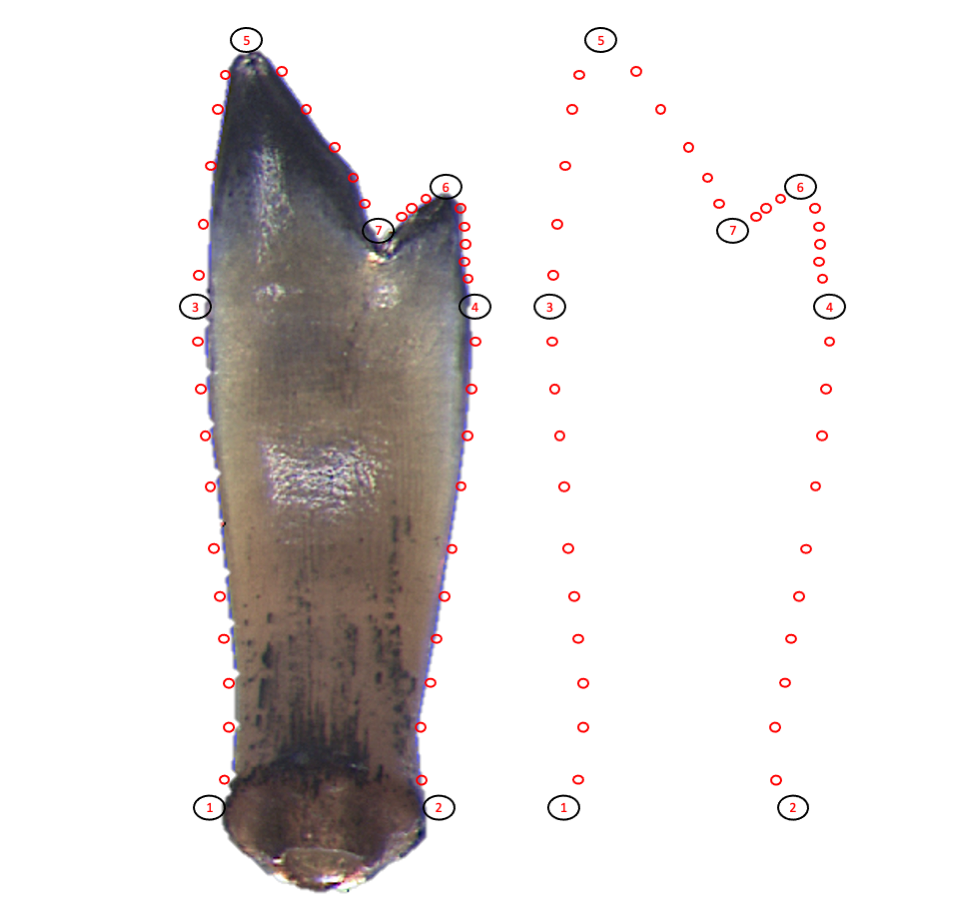


**Figure S3:** The outer oral teeth of haplochromine cichlids with red dots showing landmarks and curves.

# **Supplementary Information 3: Sediment geochronology and lake level dynamics**

The chronology was based on a total of 93 samples of terrestrial macrofossils that were radiocarbon dated using the MICADAS accelerator mass spectrometry (AMS) system at the Laboratory for the Analysis of Radiocarbon with AMS (LARA) at the University of Bern [^9^](https://paperpile.com/c/wnejhS/ZOVmv). The terrestrial plant macrofossil ages were used to construct three independent models with 95% (2σ) probabilities using rbacon [^10^](https://paperpile.com/c/wnejhS/Zl8oB) in R software and the IntCal20 calibration curve [^11^](https://paperpile.com/c/wnejhS/DJruc). The biostratigraphy of each site was used to cross-check the resulting ages [^12^](https://paperpile.com/c/wnejhS/MsFtG). The age model for LV3 was based on ^14^C dates from 26 samples (23 of charcoal, 3 bulk sediment) following the same approach as [^12^](https://paperpile.com/c/wnejhS/MsFtG).

**
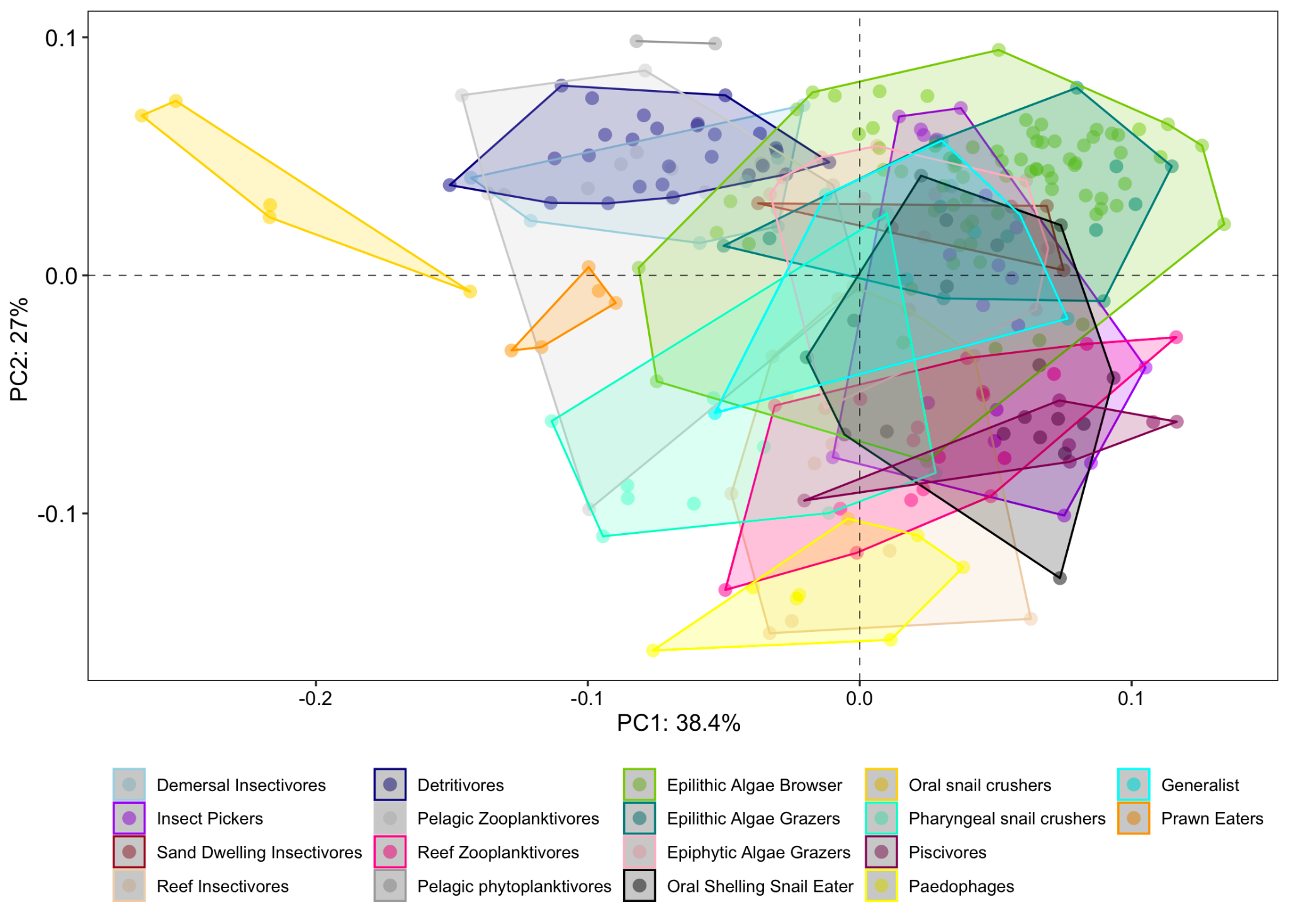
**

**Figure S4:** Principal component analysis showing patterns of morphospace grouping and occupation by the modern Lake Victoria trophic guilds.


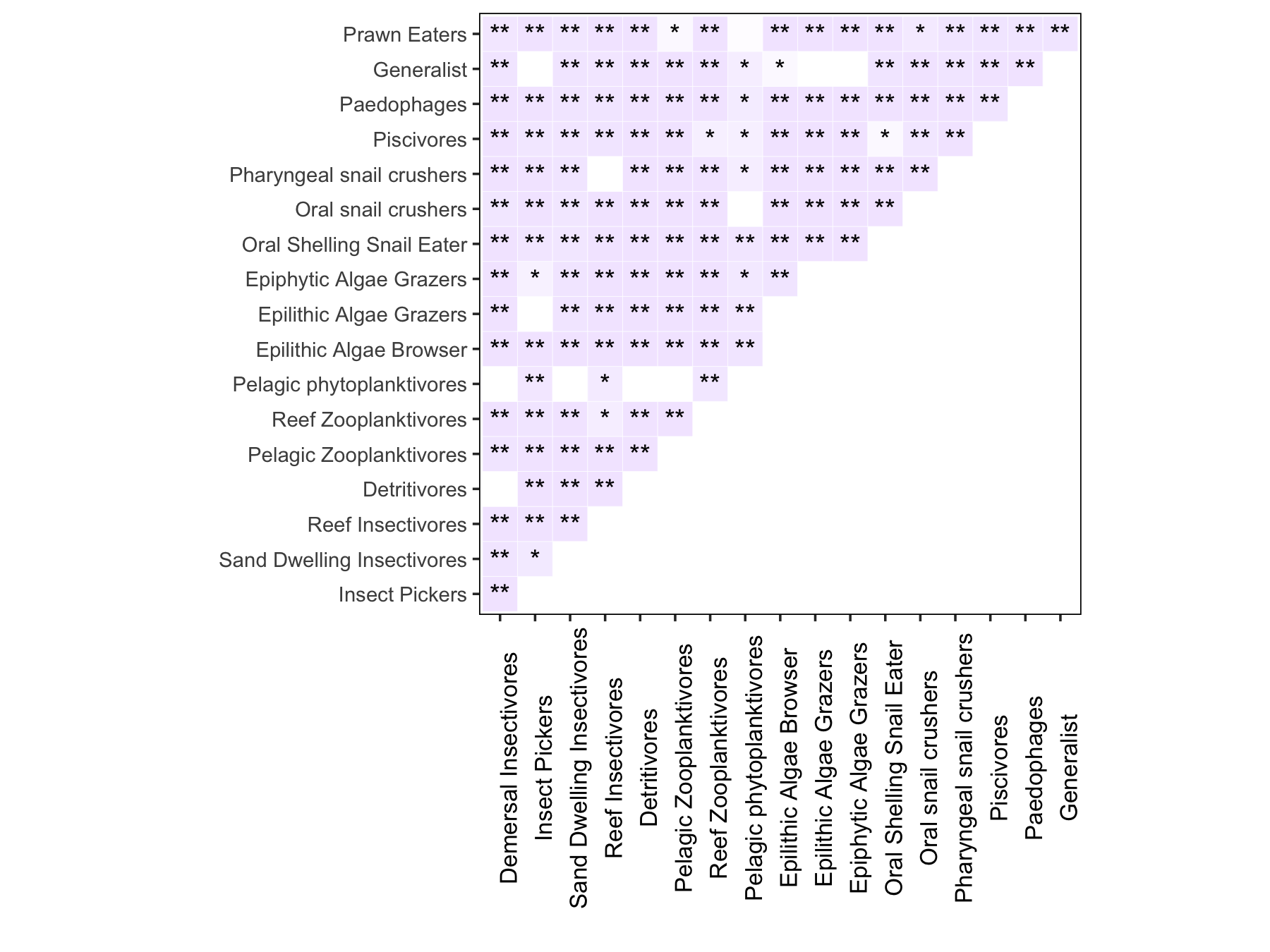


**Figure S5:** The matrix of statistical significance of pairwise differences between trophic guilds from principal components analysis of the modern LV radiation reference teeth using perMANOVA. The significant P-values are represented by the asterisks with symbols **P* ≤ 0.05, ***P* ≤ 0.01 and no asterisk is shown when relationships are not significant (P ≥0.05).


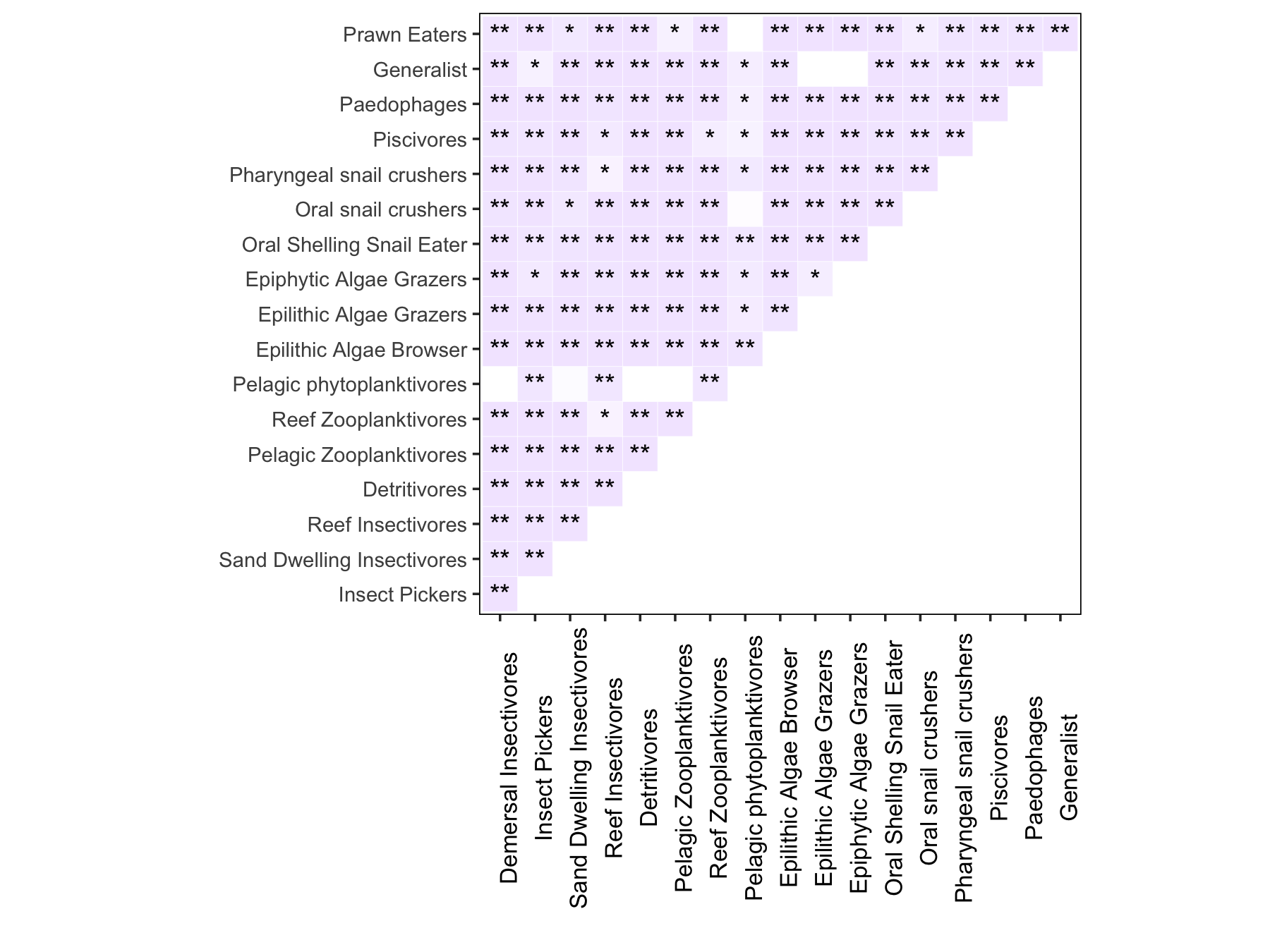


**Figure S6:** The matrix of statistical significance of pairwise differences between trophic guilds from canonical variance analysis of the modern LV radiation reference teeth using perMANOVA. The significant P-values are represented by the asterisks with symbols **P* ≤ 0.05, ***P* ≤ 0.01 and no asterisk is shown when relationships are not significant (P ≥0.05 ns).


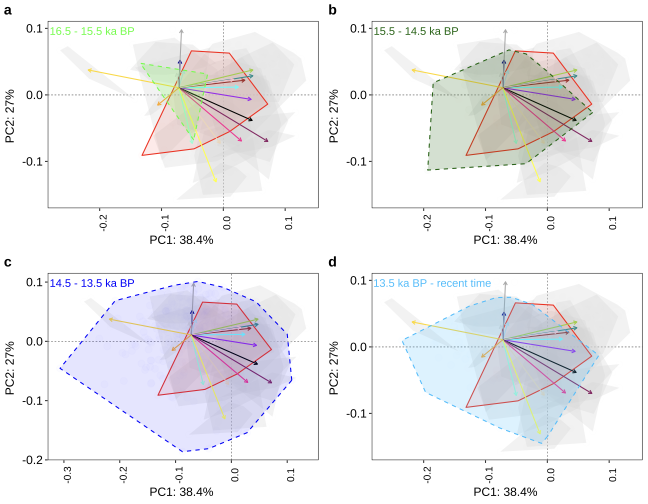


**Figure S7:** The emergence of trophic groups in the Lake Victoria cichlid fish radiation, where fossils are binned into time windows and projected into the morphospace of the modern radiation with arrow vectors representing the direction and distance of divergence from the ancestral condition for each trophic group. Reconstruction using principal component analysis (A-D) and all vectors in all time windows start from the centroid of the fossil wetland generalist that colonized the early wetland and occupied it for at least 1000 years (~16.5–15.5 ka BP) before it diversified and extended to the centroids of each modern guild. The dashed polygons are the fossil-based estimate of radiation morphospace occupied in each time window superimposed on polygons representing modern radiation, and the red polygon is the phylogenomic sister species *Astatotilapia nubila*.


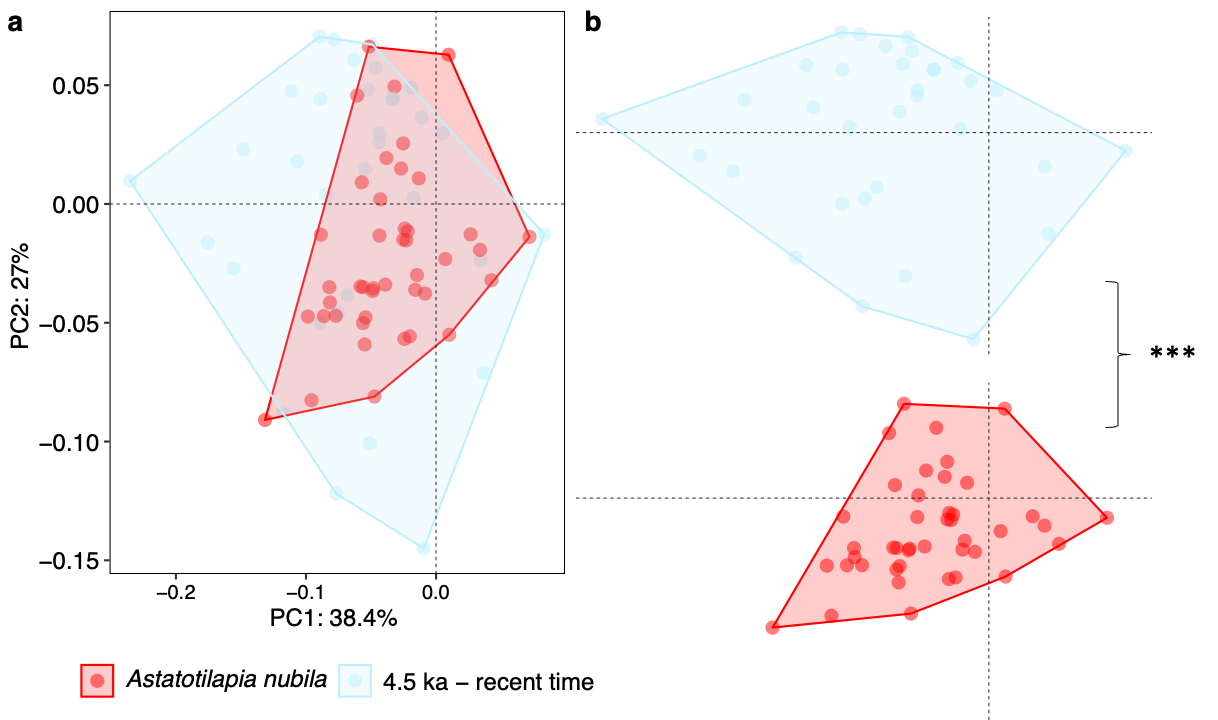


**Figure S8:** A principal component analysis of the fossils from the deep lake assemblage (~4.5 ka BP – recent time) and the modern teeth of the swamp-dwelling sister species of the radiation (*Astatotilapia nubila*), with the two groups (a) overlapped and (b) non-overlapped. The asterisk *** (P-value  ≤ 0.001) denotes the statistical significance of differences in morphological disparity between *Astatotilapia nubila* and the deep lake assemblage (4.5 ka BP – recent time).

# **Table S1:** Sequential disparity analysis using dispRity package [^13^](https://paperpile.com/c/Q961OV/41TRv) from one-time window to the next (shown as shape analysis) using a PCA morphospace defined by reference teeth and fossils projected into the morphospace, and the volume of morphospace occupied.

#
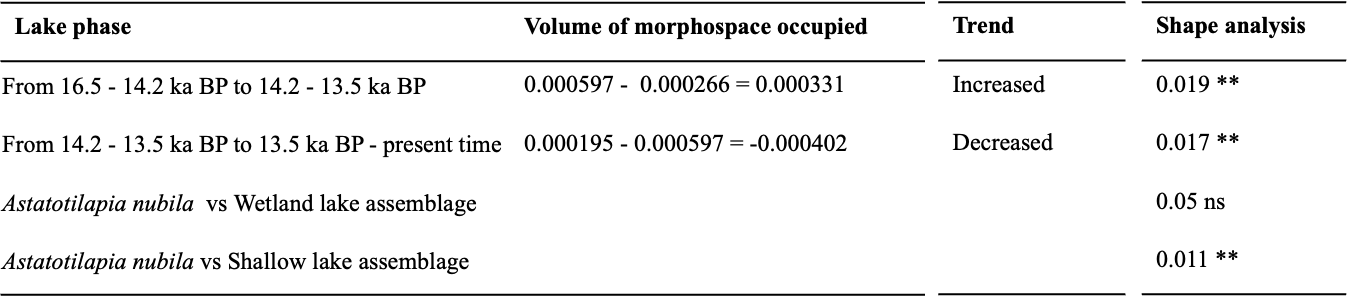


# The asterisks represent the significant P-values (**P* ≤ 0.05 and ***P≤ 0.001).

The volume change is calculated as: volume_change_i_​ = volume_i_​−volume_i−1_​

# **Supplementary References**

1. [Seehausen, O. *Lake Victoria Rock Cichlids: Taxonomy, Ecology and Distribution*. (Verduyn Cichlids, 1996).](http://paperpile.com/b/wnejhS/45n5Y)

2. [Greenwood, P. H. Cichlid fishes of Lake Victoria, east Africa : the biology and evolution of a species flock. *Bull. British Mus. Nat. Hist. (Zool.) Suppl.* **6**, 1–134 (1974).](http://paperpile.com/b/wnejhS/bSD75)

3. [Witte, F. & Van Oijen, M. J. P. *Taxonomy, Ecology and Fishery of Lake Victoria Haplochromine Trophic Groups*. (Nationaal Natuurhistorisch Museum Leiden, Netherlands, 1990).](http://paperpile.com/b/wnejhS/83Bms)

4. [Seehausen, O. & Bouton, N. The community of rock-dwelling cichlids in Lake Victoria. *Bonn. Zool. Beitr.* **47**, 301–312 (1998).](http://paperpile.com/b/wnejhS/IJVoU)

5. [Bouton, N., Os, N. & Witte, F. Feeding performance of Lake Victoria rock cichlids: testing predictions from morphology. *J. Fish Biol.* **53**, 118–127 (1998).](http://paperpile.com/b/wnejhS/8fFFB)

6. [Witte, F. *et al.* The destruction of an endemic species flock: quantitative data on the decline of the haplochromine cichlids of Lake Victoria. *Environ. Biol. Fishes* **34**, 1–28 (1992).](http://paperpile.com/b/wnejhS/vODyr)

7. [Witte, F., Van Oijen, M. J. P. & Witte-Maas, E. L. M. An Introduction To Ecological and Taxonomic Investigations On the Haplochromine Cichlids From the Mwanza Gulf of Lake Victoria. *Neth. J. Zool.* **31**, 149–174 (1980).](http://paperpile.com/b/wnejhS/goSO3)

8. [Hamilton, S. E. *et al.* High-resolution bathymetries and shorelines for the Great Lakes of the White Nile basin. *Sci Data* **9**, 642 (2022).](http://paperpile.com/b/wnejhS/dMsYC)

9. [Szidat, S. *et al.* ^14^C Analysis and Sample Preparation at the New Bern Laboratory for the Analysis of Radiocarbon with AMS (LARA). *Radiocarbon* **56**, 561–566 (2014).](http://paperpile.com/b/wnejhS/ZOVmv)

10. [Blaauw, M. & Andrés Christen, J. Flexible paleoclimate age-depth models using an autoregressive gamma process. *ba* **6**, 457–474 (2011).](http://paperpile.com/b/wnejhS/Zl8oB)

11. [Reimer, P. J. *et al.* The IntCal20 Northern Hemisphere Radiocarbon Age Calibration Curve (0–55 cal kBP). *Radiocarbon* **62**, 725–757 (2020).](http://paperpile.com/b/wnejhS/DJruc)

12. [Temoltzin-Loranca, Y. *et al.* A chronologically reliable record of 17,000 years of biomass burning in the Lake Victoria area. *Quat. Sci. Rev.* **301**, 107915 (2023).](http://paperpile.com/b/wnejhS/MsFtG)

13. [Guillerme, T. dispRity: A modular R package for measuring disparity. *Methods in Ecology and Evolution* (2018).](http://paperpile.com/b/Q961OV/41TRv)
